# Valorization of Food Waste into Single-Cell Protein: An Innovative Technological Strategy for Sustainable Protein Production

**DOI:** 10.3390/microorganisms12010166

**Published:** 2024-01-13

**Authors:** Patrick T. Sekoai, Yrielle Roets-Dlamini, Frances O’Brien, Santosh Ramchuran, Viren Chunilall

**Affiliations:** 1 Biorefinery Industry Development Facility, Council for Scientific and Industrial Research, Durban 4041, South Africa; vchunilall@csir.co.za; 2 Bioprocessing Group, Council for Scientific and Industrial Research, Pretoria 0001, South Africa; yroets@csir.co.za (Y.R.-D.); frollinson@csir.co.za (F.O.); sramchuran@csir.co.za (S.R.); 3 School of Life Science, University of KwaZulu-Natal, Durban 4041, South Africa; 4 Discipline of Chemical Engineering, University of KwaZulu-Natal, Durban 4041, South Africa

**Keywords:** single-cell protein, food waste, food security, sustainability, circular bioeconomy

## Abstract

The rapidly increasing population and climate change pose a great threat to our current food systems. Moreover, the high usage of animal-based and plant-based protein has its drawbacks, as these nutritional sources require many hectares of land and water, are affected by seasonal variations, are costly, and contribute to environmental pollution. Single-cell proteins (SCPs) are gaining a lot of research interest due to their remarkable properties, such as their high protein content that is comparable with other protein sources; low requirements for land and water; low carbon footprint; and short production period. This review explores the use of food waste as a sustainable feedstock for the advancement of SCP processes. It discusses SCP studies that exploit food waste as a substrate, alongside the biocatalysts (bacteria, fungi, yeast, and microalgae) that are used. The operational setpoint conditions governing SCP yields and SCP fermentation routes are elucidated as well. This review also demonstrates how the biorefinery concept is implemented in the literature to improve the economic potential of “waste-to-protein” innovations, as this leads to the establishment of multiproduct value chains. A short section that discusses the South African SCP scenario is also included. The technical and economic hurdles facing second-generation SCP processes are also discussed, together with future perspectives. Therefore, SCP technologies could play a crucial role in the acceleration of a “sustainable protein market”, and in tackling the global hunger crisis.

## 1. Introduction

Rapid population growth, climate variations, water shortages, and reduction in the availability of agricultural land have contributed to a surge in sustainable protein-rich foods [1]. According to data from the Food and Agriculture Organization (FAO) of the United Nations, there are more than 1 billion people around the world who suffer from malnutrition; if such a problem is not properly addressed, this number is expected to double in the next decade [2]. In sub-Saharan Africa, the problem of malnourishment is alarming, as the number has risen from 5.5 to 30 million over the last decade [3]. This was also corroborated by a study that showed that global food supplies must at least increase from 70% to 100% to reduce global hunger and malnutrition, especially in underdeveloped nations where food insecurity remains a serious concern [4]. 

The high reliance on animal-derived proteins presents several drawbacks, such as (i) contribution to greenhouse gas emissions; (ii) requirements for large amounts of water and land to grow and maintain the cattle/livestock; (iii) high costs of animal feeds; and (iv) the fact that half of the world’s fisheries have been depleted due to overfishing and anthropogenic activities, all of which further exacerbate the problem of food insecurity [5,6,7].

The literature has also shown that global meat (protein-rich source) consumption will be ~300 million tons per annum by the year 2050 [6]. This implies that other alternative sources of proteins will be essential to meet the ever-increasing demands. For this reason, modern food production approaches that embrace sustainability are receiving widespread attention amongst various stakeholders, including the scientific community, food industries, and policymakers. These environmentally conscious approaches are seen as viable solutions to some of the pressing issues highlighted above. One of the alternative strategies that has been proposed in the last two decades is the use of plant-based proteins [8,9]. Although they possess many socioeconomic benefits, they require a lot of cultivable land and water, as well as the use of chemical fertilizers, which will become unsustainable as the world strives to meet the high global demand for protein [10]. To overcome this bottleneck, the scientific community must develop robust and scalable technologies with societal, economic, and environmental relevance, particularly those that are embedded within the circular bio/economy framework. 

Recent advancements in bioprocessing and synthetic biology have expanded the use of microorganisms, and these fields are now exploited to fulfill global dietary needs such as second-generation microbial protein production, which involves upcycling various biowastes to protein-rich biomass in the form of SCP [1]. The scientific community is also advocating the use of the biorefinery concept to promote circular bio/economy practices by enabling the production of multi-product chains (SCP and other biobased products) through a network of microbial-driven pathways that leads to “zero waste” in comparison to the classical waste management strategies [1]. In addition, the valorization of biowastes, particularly food waste, into high value-added products such as single-cell proteins (SCPs), has been proposed as a novel and environmentally-benign technology that could be used to economically produce protein-rich feeds, which could serve as protein supplements for human and animal foods [11]. SCPs have all the remarkable attributes to serve as an alternative protein, as they have a high protein content (30–70%) and are low in fat; the operational costs are reduced if biowastes are used; the recovery of SCPs is relatively simple; and SCPs use little water and have a low carbon footprint [12]. 

**Figure 1:**
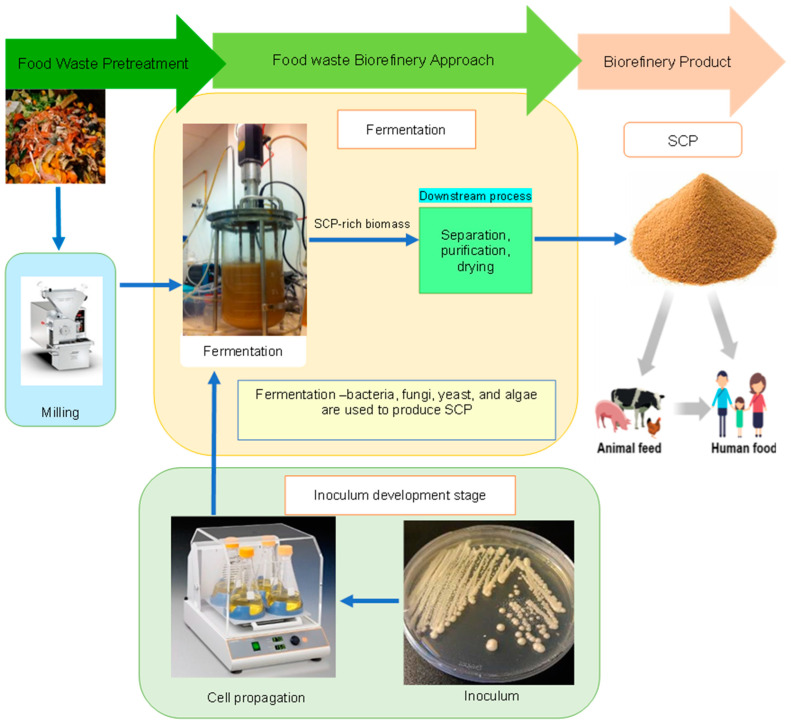
A conceptualized illustration of an SCP process from food waste.

As our society is becoming more health-conscious, a shift towards healthier protein-based sources such as SCPs is imperative, as this promotes a healthy lifestyle and/or reduces illnesses in consumers, while addressing other concerns such as animal welfare and environmental issues. A schematic diagram depicting the SCP process from food waste is presented in Figure 1.

### Significance of Study and Contribution to the Body of Knowledge

Over the past decades, there has been a growing scientific body of knowledge on the SCP process due to its health, environmental, and economic benefits as highlighted earlier. An emphasis is now being placed on the use of 2G feedstocks, such as food waste, to make this technology economically viable while alleviating environmental pollution through circular bioeconomy strategies. The outcomes obtained from the “waste-to-protein” innovations could lead to various technological breakthroughs coupled with a myriad of economic opportunities [12]. Therefore, it is imperative to communicate these R&D endeavors with both the scientific and industrial communities, as this will fast-track the industrialization of the SCP processes derived from 2G substrates. Moreover, this will be key in fostering the United Nations’ Sustainable Development Goals (SDGs), especially in the supply of healthy nutritious foods (SDG 2) like SCPs using novel sustainable technologies, particularly those SCP processes that use food waste as the main substrate. 

Against this background, this research explores a novel and eco-friendly approach that is applicable in the production of SCPs that use readily available and nutritionally-rich substrates such as food waste. This review elucidates the different microbial cell factories used in the production of SCPs, alongside the fermentation modes used in SCP production. In addition, the key operational setpoint parameters that affect the recovery of SCPs are reviewed. It also shows how the biorefinery concept could be used to accelerate R&D in “waste-to-protein” innovations. A short section that discusses the South African SCP scenario (market size, SCP start-ups/companies, etc.) is also included. The review concludes with some technological barriers that need to be addressed to advance SCP technology, particularly in developing nations like South Africa. It is hoped that this review will not only present the status quo in SCP production but also demonstrate (i) how to create/unlock economic value from biowastes; (ii) how the circular bio/economy concept could be achieved using the SCP process; and (iii) how environmental remediation could be mitigated using readily available feedstocks such as food waste.

## 2. Shedding Light on SCP

From a food technology standpoint, SCP simply refers to the protein-rich biomass that is produced through fermentation processes [13,14]. Therein, SCPs are produced using a wide array of microorganisms, including bacteria, yeasts, fungi, and microalgae [15]. The term SCP was first proposed in the 1960s by MIT scientists [16], and it was popularized by major commercial brands such as Quorn™ (produced by Marlow Foods), Marmite^®^ (produced by Unilever), *Spirulina* (produced by Cyanotech Corporation), etc., which are produced using fungus (*Fusarium venenatum*), yeast (*Saccharomyces cerevisiae*), and microalgae, respectively [17]. In contrast to animal-derived proteins and plant-based proteins, microbial cell factories present several socioeconomic benefits as alternative suppliers of proteins, as they have minimal requirements for land and water, coupled with their role in the field of circular bioeconomy. SCPs are also used as an alternative/cheap dietary protein source for animal feed, i.e., aquaculture and livestock [18]. 

Interestingly, most microbes are characterized by a high protein content. For instance, algal species (e.g., *Arthrospira platensis*, *Chlorella vulgaris*, *Dunaliella salina*, *Galdieria sulphuraria*, and *Tetraselmis chui*) have been shown to produce 40–60% of SCPs [19,20], while edible fungi can generate 30–70% of SCPs [16]. Bacteria and yeast are also common in SCPs due to (i) their high SCP yields (50–80%); (ii) metabolic robustness and versatility; (iii) high-throughput metabolic data that enables scientists to acquire deep insights into their physiological conditions; (iv) sufficient literature regarding their cultivation and/or engineering; (v) and their ability to assimilate various carbon sources including biowastes [12]. SCP is produced using submerged or solid-state fermentation; the former process is commonly used in the literature due to its simplicity and the ability to use various bioreactors, with most microbes tending to grow well in liquid mediums; the latter process is preferred owing to its high SCP yields, high substrate conversion, and low operational costs [21,22]. 

However, bacterial-derived SCP and fungal-derived SCP also come with their limitations, as these microbial cell factories produce a high RNA content (15–16% on a dry basis), which is toxic to humans [23]. This problem is usually addressed by subjecting the protein-rich biomass to heat treatment (e.g., 60–70 °C for 30 min), although the economic implication of this downstream process needs to be examined [16]. Recent advances in recombinant DNA have also allowed scientists to concomitantly produce SCP with other valuable compounds using genetically engineered strains, thus creating new industrial value chains [24]. For the scope of this review, this research solely focused on the production of SCP using food waste as a carbon source. Moreover, the setpoint parameters and other crucial factors governing the overall process performance will be broadly discussed in the next chapters of this review.

## 3. Availability of Food Waste as a Sustainable Feedstock for SCP Production

With a significant emphasis being placed on the use of second-generation feedstocks in order to avert the “Food vs. Fuel” conundrum, there is also a paradigm shift in the generation of SCP. Scientific researchers are tapping into non-edible and cheap substrates to make the process sustainable and industrially-competitive, while reducing the environmental burdens caused by food waste.

Food waste is by far the most widespread and readily available feedstock that can be used for the attainment of sustainable and cost-competitive SCP technology. Rapid population growth, improved standards of living, and high levels of industrialization are some of the main contributing factors that lead to the continual increase in food waste [25]. The United Nations Environment Program’s Food Waste Index estimates that around 1 billion tons of food waste is globally produced each year, to which industrialized nations contribute a large fraction [26]. In the past, these nations relied heavily on traditional and unsustainable food waste treatment methods (landfilling and incineration), but stringent waste disposal regulations are compelling them to replace these with eco-friendly food waste handling/treatment approaches—these technologies embrace the concept of a circular bioeconomy, and developed nations like the EU are pioneering this research area [27]. 

Unfortunately, food waste still poses a threat to both humans and ecological habitats in developing countries like South Africa, as these nations lack adequate waste management technologies [28,29]. A study by the Council for Scientific and Industrial Research (CSIR) revealed that approximately 10.3 million tons of food waste are produced each year in South Africa, and this value will still increase in the coming years due to the abovementioned contributors [30]. This continuous increase in food waste is alarming when considering that developing nations like South Africa only recycle 10% of their waste (including food waste), while the remaining 90% is disposed of in landfills or is incinerated, causing an array of environmental issues [31]. Recently, the Department of Environmental Affairs of South Africa introduced strict environmental policies that aim to completely eradicate organic waste disposal by the year 2027 [32], implying that local industries and scientists need to respond promptly and develop novel biowaste valorization technologies to resolve this societal issue. This will save the South African government a lot of money, as millions of rands are spent each year on waste management strategies. 

A review by Dahiya et al. [25] showed that the use of food waste in microbial biorefinery-based technologies could provide major breakthroughs in the field of biobased economies, as this can lead to the production of diverse market-based products such as biomaterials, biofuels, biochemicals, bio-additives, and biofertilizers, including SCP, via a “cascade” and/or “one-pot” fermentation approach. Regarding its nutritional composition, food waste is a desirable source of SCP, as it has low lignin content compared to agro-wastes, which require extensive and often expensive biomass fractionation methods to extract the C_5_ and C_6_ fermentable sugars [33]. Food waste consists of other essential growth factors such as starch, cellulose, proteins, and lipids [34,35].

## 4. Nutritional Composition of Food Waste and Its Suitability for SCP Production

Several studies have been undertaken in the field of microbial processing to ascertain the nutritional composition of food waste derived from the industrial, agricultural, commercial, and municipal sectors [36,37,38]. Therefore, this review builds upon this existing scientific data to reiterate the significance of food waste as a sustainable substrate that can be used for the advancement of SCP processes. Food waste has the essential organic constituents to promote the growth of diverse microbes during fermentation processes such as those discussed above. 

A study by Zhang et al. [39] revealed that food waste derived from agricultural, industrial, food retail, and municipal sectors is an optimal substrate, as it has balanced nutrients for the cultivation of different microbial cell factories; it has a moisture content of 74–90%, a volatile solids to total solids ratio (VS/TS) of 80–97%, and a carbon to nitrogen ratio (C/N) of 14.7–36.4. Elsewhere, it was reported that food waste is well-suited for various biochemical processes, as it has vast amounts of micro-nutrients (e.g., iron, magnesium, manganese, copper, zinc, vitamin B6, vitamin C, vitamin K, selenium, etc.) and macro-nutrients (e.g., fiber, minerals such as iron and potassium, and vitamins such as vitamin B9, vitamin E, vitamin A, vitamin K, riboflavin, etc.) which act as stimulatory agents to microbial consortia [40]. There are slight variations in these findings, as other factors also contribute to food components (e.g., geographic location, climate, processing of food waste, storage conditions, etc.); however, most studies coincide with these results, as shown in similar studies conducted in other regions such as Asia [41,42], Europe [43], Africa [44], and North and South America [45]. From a techno-economic standpoint, fruit and vegetable wastes are deemed suitable for SCP due to their high composition of monomeric sugars (glucose and sucrose), low lignin content, biodegradable nature, ability to be assimilated by a wide spectrum of microorganisms, and high accessibility [46,47]. It has also been shown that fruit wastes such as pineapple peel, orange peel, potato peel, banana peel, and carrot pulp increase the SCP yields due to their high composition of sugars that ranges from 54.17 to 83% [48,49]. 

In addition, these carbon sources may reduce processing costs by 30–50%, since they require less energy-intensive pretreatment methods as opposed to agro-wastes, which are rich in lignin [50]. On the contrary, there is skepticism regarding the use of agro-food waste such as corn cobs, rice straw, bean husks, and bagasse, as they mainly consist of cellulose (35–50%), hemicellulose (25–30%), and lignin (25–30%), which must undergo extensive fractionation steps before being used in SCP production processes; thus, these sources are not economically viable [51]. 

Recent studies that discuss the use of food waste as a substrate in SCP showed promising results. For example, maximum protein contents that varied from 27 to 36% were obtained during the production of SCP using bread waste as a carbon source and the fungus known as *Rhizopus delemar* [52]. In another study, fungal species of *Aspergillus oryzae*, *Neurospora intermedia*, and *Rhizopus oryzae* were used to produce high protein fractions of 44.7%, 57.6%, and 50.9%, respectively, using vinasse as a substrate [53]. Meanwhile, a pea processing byproduct was used to cultivate fungal strains of *Fusarium venenatum*, *Aspergillus oryzae*, *Neurospora intermedia*, *Monascus purpureus*, and *Rhizopus oryzae* [53]. The results were satisfactory, as the protein content ranged from 43.14 to 55.28% without the addition of α-amylase—an enzyme used for pretreating the substrate [54]. Interestingly, a remarkable increase in protein content (46.36–59.75%) was observed upon the addition of α-amylase, which implies that it is effective in the recovery of fermentable sugars such as glucose [54]. These results correlate with other scholarly reports because edible fungus strains such as *F. venenatum* can produce up to 50% of the protein, and these biocatalysts are already used in large-scale SCP processes—an example includes the commercial Quorn™ brand, which is produced by Marlow Foods [55].

## 5. An Overview of SCP-Producing Microbes

Bacteria, microalgae, oleaginous fungi, and filamentous yeasts are potent inoculum sources that are primarily used in the biosynthesis of SCP, as shown in Table 1. These biocatalysts are covered in this section. 

### 5.1. Bacteria

The use of bacteria in SCP is well documented in the literature; these inoculum sources are ubiquitous and can assimilate a broad range of carbon sources, including food waste [56]. The use of bacteria in SCP is reported both at the lab scale and pilot scale [57], and this is due to their ability to generate high protein yields (50–80%), as explained earlier. Amongst the bacteria species, methanotrophic bacteria are widely exploited, as they can generate high SCP yields; their industrial applications in SCP date back to the 1960s [58]. In addition, they can use methane as a carbon and energy source, while metabolizing the nutrients that are available within the fermenter [58]. Methane-oxidizing bacteria such as *Methylomonas* sp. and *Methylococcus* sp. have been shown to produce up to 56% [58] and 70% [59] of SCP, respectively. The production of methane-based bacterial SCP fits well within the scope of a circular economy, as these bacteria can synthesize a wide spectrum of new-value-added compounds via the “closed-loop” biorefinery-based approach. Furthermore, a synergistic relationship is established when mixed-bacterial cultures are used; this confers numerous benefits during SCP fermentation, as specific microbial species aid in the conversion of substrates, while others boost the SCP production yields [60]. 

This technology is currently being used by SCP-producing companies such as FeedKind^®^ and Uniprotein^®^ [59]. Other genera, such as *Bacillus* sp. and *Lactobacillus* sp., have been explored in the literature, and studies that used these inocula obtained SCP yields of 84% [61] and 71% [62], respectively. To create maximum value chains from food waste, novel valorization routes targeting SCP and other value-added compounds such as biopolymers, biofertilizers, biosurfactants, biolipids, biofuels, etc., have been explored in the literature to achieve biorefinery-based strategies that also lead to “zero waste” [56]. However, the use of bacteria has been scrutinized, as these inocula are smaller in size and less dense compared to yeast and fungi, which makes it difficult to recover them [18]. They also produce high nucleic acid content, which is harmful to humans, as discussed earlier. 

### 5.2. Fungi

Fungi have been extensively exploited in SCP production studies due to their metabolic robustness, high SCP yields, and high density, and they offer an affordable downstream process [63]. Fungal-derived SCP possesses yield health benefits, i.e., it has various micronutrients such as vitamin B12, riboflavin, phosphorus, zinc, and manganese, as well as low amounts of cholesterol [64]. Furthermore, fungi are renowned for secreting large quantities of hydrolytic enzymes, which play an important role in the biodegradation of substrates, including organic wastes [65,66]. Research regarding the use of *Fusarium venenatum*, *Aspergillus niger*, and *Trichoderma* sp., is attracting a lot of scientific and industrial attention, as these fungi can generate SCP yields that range from 30 to 70%, and have moderate nucleic acid yields (7–10%) that need to be processed [67]. The success of fungal mycoprotein has also led to the development of commercial SCP brands such as Quorn^®^, Marmite^®^, and Vitamin-R^®^, which are produced by large corporations such as Marlow Foods, Unilever, and Vitam Hefe-produkt GmbH, respectively [17]. In addition, the use of food waste in fungal-derived SCP processes is advantageous, as great strides have been made in the development of enzyme-producing fungal strains (e.g., *Trichoderma*, *Aspergillus*, *Rhizopus*, *Neurospora*, etc.); these are used for the hydrolysis, saccharification, and fermentation of various biowastes, as opposed to the contemporary biomass fractionation techniques that involve the use of costly and toxic chemicals [68]. These scientific milestones have led to the discovery of the “closed loop” fungal strategies phenomenon, which facilitates the synthesis of a gamut of biobased products based on the one-pot fermentation approach, while addressing sustainability and the circular bio/economy. Fungal studies are also used to address the issues caused by agro-food industrial sidestreams by creating novel pathways for circular opportunities [68]. Wastewater produced by fruit-processing, cheese-processing, potato-processing, wheat-processing, and brewing industries can now be used for the large-scale production of SCPs, leading to the “zero-waste” concept [69]. 

The use of fungal-based biorefinery strategies is expected to create new biobased markets, as organic acids such as citric acid and gluconic acids are already produced at the industrial scale by *Aspergillus niger* [68] alongside lactic acid, which is produced by *Rhizopus* sp. [68]. Other fungal strains such as *Mortierella wolfii* [70] and *Aspergillus terreus* can also produce fatty acids when cultivated in growth media consisting of food waste [71]. Recent studies have also explored the utilization of wastewater derived from various food industries for the cultivation of *Pythium irregulare* to produce other food-related products such as long-chain polyunsaturated fatty acids (LC-PUFAs) [63]. These scientific reports underscore the importance of fungal research, as it has been demonstrated to have an impactful role in food security and environmental remediation, and such research can be implemented in new concepts such as biorefineries and the circular bioeconomy. 

### 5.3. Microalgae

Microalgal research has enabled researchers to achieve many scientific breakthroughs, as it is now also used in SCP production studies. Microalgae are well suited for human and animal consumption, as some species can yield high protein content (60–70%) as well as other essential nutrients (omega-3, omega-6, vitamin-A, -B, -C, -E, minerals, etc.) [68]. Currently, microalgae are incorporated into many food formulations, and this has enhanced their value chains. These supplements are sold as capsules and/or dried powders. The most notable microalgal-derived SCP product is *Spirulina* (*Arthrospira platensis*), a product that is sold as a protein-rich supplement [72]. It is one of the most cultivated microalgae because of its high protein content (up to 70%) when cultivated under non-limited nitrogen conditions [72]. It is also rich in other valuable compounds such as carotenoids, chlorophylls, and phycobiliproteins, which are also applied in the food industry [72]. *Spirulina* has also been shown to enhance the immune system and reduce the risks of developing illnesses such as cardiovascular disease, degenerative chronic disease, cancer, etc. [73]. As a result of its nutritional profile coupled with its health benefits, *Spirulina* has been certified by the United States Food and Drug Administration (US FDA) as safe for human consumption [74]. Other algal-based SCP producers include *Chlorella* and *Senedesmus* [75]. These algal species are useful in other applications such as wastewater treatment/waste stabilization [76]. These species were co-cultured during a bench-scale study that focused on the anaerobic digestion of food waste [77]. It was reported that the addition of two algal species not only produced a high biomethane yield (639.8 mL/gVS_added_), but also resulted in significant production of SCP (76.35%) [77]. A pilot-scale SCP study was conducted to produce SCP (43.1%) using *Senedesmus* as the inoculum, and it was discovered that the amino acid content was similar to that of soybeans [78]. It was also observed that this biomass can be used for the formulation of animal feed [78]. In addition, the use of *Senedesmus* can play a critical role in promoting a circular bio/economy, as it can produce many biochemicals and biofuels [79]. Elsewhere, a pilot-scale process was also accomplished using *Chlorella* to synthesize SCP, β-carotene, and chlorophylls [80]. Other algal species, such as *Schizochytrium*, *Haematococcus*, *Dunaliella*, and *Arthrospira*, are applicable in SCP studies because they are generally regarded as safe (GRAS) by the FDA, and their use will lead to new innovations/discoveries in the field of SCP [81]; furthermore, these species are also used for commercial purposes [82]. Recent advances in genetic engineering and molecular biology have allowed researchers to discover microalgal strains with robust metabolic traits, especially those strains that can facilitate the production of multifarious value-added products such as proteins, antioxidants, biopolymers, carotenoids, fatty acids, carbohydrates, etc., via microalgal biorefinery-based strategies. These newly developed technologies are indispensable to the attainment of circular biobased economies [83]. In these approaches, the primary extraction techniques are applied to recover the protein, followed by the secondary extraction techniques that target other valuable products [83]. A recent study showed that integrated microalgal biorefinery-based strategies can be used for beneficiating industrial sidestreams, industrial CO_2_ sequestration, and biomass valorization [84]. *Aurantiochytrium* sp. is another genus that is extensively used in food waste valorization strategies, since it produces a high content of polyunsaturated fatty acids (PUFAs), and has also been shown to produce desirable yields of protein and carbohydrates when applied in a biorefinery framework [68]. However, the use of algae requires further scrutiny, as they need many hectares of land for growth and a constant supply of nutrients.

### 5.4. Yeast

Yeast species are also used for SCP studies because of their superior nutritional yields and metabolic robustness. They can assimilate diverse carbon sources including food waste, thus making them a desirable inoculum source [12]. To embrace the concept of “waste-to-protein” through sustainable innovations, yeasts such as *Saccharomyces cerevisiae*, *Candida utilis*, *Cryptococcus curvatus*, *Kodamaea ohmeri*, *Debaryomyces prosopidis*, *Yarrowia lipolytica*, *Lipomyces starkeyi*, *Rhodotorula glutinis*, *Rhodosporodium toruloides*, etc., are widely used in SCP production due to their high protein yields (30–70%); moreover, they can be grown on food waste media [85]. Similar to fungal biorefineries, current research is exploring the use of yeast-based biorefinery strategies (i) for various biowaste valorization routes using yeast strains; (ii) to reduce the operational costs associated with the upstream and downstream processes; and (iii) to enable the upcycling of industrial sidestreams to reduce their disposal costs [85]. Yeast cells do not need to be lysed in comparison to algae. Moreover, yeast species exhibit remarkable adaptability, and their growth can be enhanced through a range of strategies. These include the careful selection of yeast strains, selection of suitable substrates, consideration of the byproducts generated during whole-cell biomass production, and the refinement of biomass processing techniques to yield a final, practical prototype. However, one of the drawbacks of using yeasts for SCP production is the high nucleic acid content. In addition, some yeast species such as *S. cerevisiae* produce undesirable/unwanted byproducts such as ethanol; therefore, it is important to understand the growth profile of species that are to be used in SCP fermentation [5]. 

## 6. Modes of Fermentation Used in SCP Production

### 6.1. Submerged Fermentation

Submerged fermentation entails the use of a liquid medium whereby the fractionated substrate is immersed in a liquid nutrient medium to enable enzymatic and/or microbial hydrolysis during the SCP process [86]. The fermentation is conducted in a bioreactor that is well suited for the cultivation of SCP-producing microbes, and it is operated under batch, semi-batch, or continuous mode to obtain the protein-rich biomass [87]. In addition, the bioreactors are operated under optimal pH, temperature, aeration, and fermentation conditions [88]. The acquired SCP is recovered through filtration or centrifugation, followed by drying the biomass.

### 6.2. Semi-Solid Fermentation

Semi-solid fermentation has been shown to exhibit remarkable results in the fields of fermentation technology and bioprocess engineering; it generates high yields, requires lower energy inputs, and uses little water [89]. However, other authors have argued its use in SCP, as this may exacerbate the operational costs due to the need for advanced biomass fractionation methods and/or the use of genetically modified microorganisms to hydrolyze the substrate [90]. Moreover, other crucial steps such as proper sterilization, suitable conditions for the upstream process, and product recovery will need to be considered when adopting this fermentation approach [91]. 

### 6.3. Solid-State Fermentation

Solid-state fermentation is widely applied in numerous biotechnological applications involving the production of feed additives, biofuels, biochemicals, etc. [92]. It is defined as a fermentation strategy that excludes the use of free water; however, the substrate must have sufficient moisture to facilitate microbial growth and enzymatic processes [93]. Solid-state fermentation dates back to ancient times when it was used for different applications [93]. This fermentation approach has attracted increasing worldwide attention due to its several benefits, i.e., it uses little energy and water, produces little wastewater, and contributes to environmental remediation [94]. This technology is also used in SCP, and researchers have observed desirable SCP outputs [95,96]. It is common in studies employing fungal species such as *Aspergillus niger*, *Candida* sp., *Trichoderma* sp., and *Saccharomyces cerevisiae*, as these inocula tend to produce protein-rich biomass [97,98]. 

**Table 1:** An overview of SCP production studies from food waste.

Food Waste	Inoculum	Fermentation Type	Protein (%)	Reference
Corn Stover	*Rhodococcus opacus*	Submerged	52.7	[99]
Olive fruit waste	*Candida lipolytica*	Submerged	69	[100]
Fruit waste	*Saccharomyces cerevisiae*	Submerged	79.14	[101]
Molasses	*Kluyveromyces marxianus*	Submerged	50.5	[102]
Food waste	*Saccharomyces cerevisiae*	Solid-state	38.43	[103]
Soy molasses	*Candida tropicalis*	Submerged	56.41	[104]
Cheese whey	*Trichoderma harzianum*	Submerged	34.21	[105]
Tomato, mango, orange, apple, and banana	*Trichoderma reesei*	Solid-state	14.06–78.17	[106]
Paddy straw, wheat straw, sugarcane, and maize straw	*Bacillus licheniformis*	Solid-state	24.56	[107]
Soybean wastewater	*Rhodobacter sphaeroides Z08*	Submerged	52	[108]
Fruit waste	*Saccharomyces cerevisiae*	Solid-state	48.32	[109]
Food waste	*Yarrowia lipolytica*	Submerged	38.8	[110]
Agri-industrial wastewaters	Purple phototrophic bacteria, algae	Submerged	60	[111]
Tofu waste	*Chlorella* sp.	Semi-solid-state	52.32	[112]
Tempeh	*Chlorella* sp.	Semi-solid-state	52	[112]
Cheese whey	*Chlorella* sp.	Semi-solid-state	15.43	[112]
Food waste	*Saccharomyces cerevisiae*, *Candida utilis*, *Yarrowia lipolytica*	Solid-state	25.14	[113]
Waste bread	*Neurospora intermedia*	Submerged, solid-state	27–33	[114]
Potato protein liquor	*Neurospora intermedia*, *Rhizopus oryzae*, *R. oligosporus*, *R. delemar*, *Aspergillus oryzae*,	Submerged	53	[115]
Stale bread and brewers’ spent grain	*Neurospora intermedia*, *Rhizopusoryzae*	Solid-state	21.1	[116]
Bread residues	*Neurospora intermedia*, *Aspergillus oryzae*	Submerged	45	[117]
Rice straw	*Chlorella sorokiniana*	Submerged	40–45	[118]
Coffee wastewater	*Candida sorboxylosa*	Submerged	64.4	[119]
Fish processing sidestreams	*Rhizopus oryzae*	Submerged	33–62	[120]

## 7. Parameters Governing the Yields of SCP

In addition to the carbon source, the technology of SCP is governed by various physico-chemical parameters such as the pH of the fermentation medium, temperature, nitrogen source, inoculum size, oxygen transfer, etc. These setpoint variables are usually examined, one at a time, in scientific literature to acquire deeper insights into their individual roles in SCP-producing pathways and SCP yields during the downstream process [121]. For this reason, the individualistic impact of the setpoint parameters governing SCP productivity is discussed in this section. 

Amongst the parameters that are documented in the literature, the temperature remains one of the most studied setpoint variables in the literature, since it affects the (i) growth and activity of microorganisms, (ii) feedstock utilization, and (iii) metabolic pathways targeting SCP [122,123]. A temperature range of 25–30 °C is commonly used in SCP production [21]. However, other fermentation studies reported optimal temperatures of 35 °C [124] and 37 °C [125]. It is imperative to understand the effect of temperature on the growth of inocula, since bacteria, fungi, and microalgae have varying optimal temperatures. For example, it was revealed that *Candida utilis* enhances the SCP yield when the temperature is increased from 25 to 35 °C [126]. Meanwhile, studies employing other microbial species such as *Saccharomyces cerevisiae* and *Bacillus subtilis* recorded optimal temperatures of 30 °C [127] and 37 °C [125], respectively. Other SCP producers such as *Fusarium venenatum* grow well at a temperature range of 28–30 °C [128,129]. 

pH is another key setpoint variable that should not be overlooked in SCP studies, as it affects enzymatic pathways, proliferation of inhibitors, and hydrolysis rates [130,131]. Contradicting values have been reported in the literature regarding the optimal pH for SCP fermentations. This is attributed to the diverse microbial strains used, as these inocula have varying physiological requirements. Nevertheless, most SCP fermentation studies use a pH ranging from 3.5 to 7.0 [21]. SCP studies involving the use of bacteria tend to thrive under moderate/alkaline pH values [96], whereas some yeasts and fungi can still thrive under acidic conditions (pH 3.5–5.5) [132,133]. 

The attainment of high SCP yields also depends on the nitrogen source. The nitrogen source is obtained from nitrogen-containing chemicals such as urea, ammonium salts, nitrate, and nitrogen, which are obtained from different organic feedstocks [134]. Like the carbon source (food waste), the nitrogen source needs to be used in an optimal range to obtain desirable yields. Somda et al. [135] studied the effect of supplementing different nitrogen sources (peptone, ammonium sulphate, ammonium nitrate, and yeast extract) on SCP production using *Candida utilis* FJM12. The authors achieved high protein-derived biomass after 72 h when using peptone (6.48 g/L) as a nitrogen source, followed by ammonium sulphate (5.74 g/L), and ammonium nitrate (3.77 g/L) [135]. In each experimental run, 1% (*w*/*v*) of nitrogen was used as a supplementary growth medium [135]. In another similar study, it was reported that 0.6% (*w*/*v*) of nitrogen was suited for the cultivation of various yeast strains such as *S. cerevisiae*, *K. marxianus*, *T. cremoris*, and *M. hiemalis* [136]. 

Nonetheless, research is currently focused on the use of nutrients from biowastes. It has been shown that nitrogen derived from biowastes can contribute to the advancement of SCP process, since these are readily available and inexpensive compared to nitrogen derived from chemicals. Consequently, most fermentation studies are currently exploring the use of various biowastes attained from various sectors such as agro-processing, food, and municipal industries. In a recent study, it was shown that the supplementation of corn-steep liquor resulted in a significant increase in SCP [137]. Therein, the authors used the simultaneous saccharification and fermentation strategy with a cell productivity of 0.23 g L^−1^ h^−1^, using the *Candida intermedia* FL023 yeast strain [137]. 

Aeration is another crucial growth factor that should be taken into consideration in SCP studies; it is needed for the growth and activities of microbial cells [138]. A study that investigated the effect of aeration on SCP yield using *Candida utilis* reported 1 vvm as an optimum aeration rate [139]. This was also corroborated by a study that used different yeast strains; the highest SCP yield was achieved at 1 vvm when using *K. lactis*, *S. cerevisiae*, *K. fragilis*, and *K. marxians* [140]. Fermentations that used bacteria such as *Bacillus*, *E. coli*, *B. coagulans*, *B. stearothermophilus*, and *B. licheniformis* were shown to thrive under aeration of 0.5–1.5 vvm [141].

## 8. Single-Cell Protein Production in South Africa 

### Current Status, Market Analysis, and Future Outlook

South Africa is in dire need of sustainable protein production technologies, since its continuously growing population and unfavorable climatic conditions negatively impact the supply of current protein production sources (animal- and plant-based proteins). Although it is not yet comparable to that from Western nations, research about single-cell protein/mycoprotein is receiving widespread recognition/acceptance in South Africa, since food industries, scientists, and the government are committed to augmenting the country’s protein supply via eco-friendly and sustainable approaches. Moreover, this technology will help address some of the challenges derived from the high reliance on traditional protein sources (animal- and plant-based protein), as these require substantial amounts of land and water, are affected by seasonal changes, and are costly, as discussed earlier. Also, as a semi-arid and environmentally-strained country, South Africa needs to stabilize its nutritive protein sources [142]. Nonetheless, most SCP products that are sold for both human consumption and livestock cultivation are imported from international corporations based in Europe, North America, and Asia. Currently, Unilever remains as the major SCP-producing food company—Marmite^®^ is the most popular yeast product in South Africa, and is widely used as a protein spread [17]. 

Recent reports on food consumption trends reveal that most South Africans are increasingly becoming health-conscious, and there is a drive for alternative protein sources. The country is ranked 22nd and 23rd in the world for veganism and vegetarianism, respectively [143]. South Africa contributes about 57% of Africa’s total plant protein market—this industry is estimated to be around USD 400 million [144]. Hitherto, beans, soy, rice, and wheat remain the most popular plant-based protein sources in the country, and the drive for alternative and healthy protein sources is envisioned to positively impact the advancement of SCP in South Africa [145]. 

It is worth mentioning that the aforementioned protein sources are mostly used in animal feed; some of these are imported to ensure that there is sufficient supply. The animal feed market in South Africa generated around ZAR 50 billion in sales in 2021, and employs more than 17,000 people [146]. Despite its success, the Animal Feed Manufacturers Association (AFMA) of South Africa has indicated that the long-term profitability/success of the industry will be reliant on the supply of cheap and sustainable feedstocks [147]. Hence, the intensification of second-generation microbial protein technologies such as SCP may play a significant role in addressing this bottleneck, as overabundant biowastes such as food waste alongside an array of microbial cell factories could be used to economically produce protein and create new value chains [148]. 

From an R&D standpoint, several SCP-related research studies have been undertaken by the country’s leading academic institutions such as the University of Cape Town, Stellenbosch University, University of Free State, and the University of Pretoria, among others, including the country’s leading science councils such as the Council for Scientific and Industrial Research. Although most of these SCP-related projects have just commenced and are mostly conducted at low TRLs, the obtained results will help scientific researchers, food industries, and policymakers make informed decisions when investing in robust and commercially-appealing SCP technologies in the future [149]. 

## 9. Economic Analysis

Apart from the environmental benefits of transforming food waste into SCPs through sustainable innovations, scientists and industries are aiming for biobased technologies with positive economic outcomes, so that these processes can serve as alternatives to fossil-based technologies, as substantiated by the United Nations’ SDGs. Therefore, several studies have been undertaken in recent years to acquire deeper insights into the economic implications of developing SCP technologies that are derived from 2G feedstocks such as food waste. To achieve this, researchers use a scientific tool known as techno-economic analysis (TEA) to evaluate the economic performance of SCP processes. Based on the study of Aggelopoulos et al. [150], it is economically-viable to produce SCPs using food waste as raw materials to account for 35–55% of the manufacturing costs. Furthermore, the use of low-lignin-containing (biodegradable) feedstocks such as food waste will reduce the operational costs, as significant expenditure is required to purchase expensive commercial enzymes and biomass fractionation reactors [151]. As shown in Section 5, an emphasis is also placed on SCP processes that embrace the biorefinery concept of producing “zero waste” while diversifying the product portfolio—SCP is co-produced with other valuable compounds. It was recently shown that it is more profitable to synthesize SCP via anaerobic digestion, since this produces biogas and biodigestates compared to traditional approaches that focus solely on biogas as the biobased product [152]. Other TEA studies have shown the economic feasibility of having biorefineries that target multiproduct value chains (SCP, natural fibers, and methane) using agro-industrial effluents [153]. A more recent study by Vlaeminck et al. [154] demonstrated that third-generation biorefineries harnessing industrial off-gases could be another economically feasible approach for biobased SCP innovations, as this strategy led to cost reductions of 4.15 to 2.78 USD/kg. 

## 10. Conclusions and Suggestions for Future Research

This review explored the feasibility of using food waste as a sustainable feedstock for the advancement of SCP processes. Furthermore, it discussed the SCP studies that exploit food waste as a substrate alongside the inocula (bacteria, fungi, yeast, and microalgae) that are used. The operational setpoint conditions affecting SCP yields and SCP fermentation routes were reviewed as well. This review also demonstrated how the biorefinery concept is implemented in the literature to improve the economics of “waste-to-protein” innovations, since this leads to the establishment of multiproduct value chains; moreover, South Africa’s efforts towards the attainment of alternative protein sources were discussed.

As shown in the United Nations’ SDG 2, one of the main challenges of the 21st century is providing affordable and nutritious food to the continuously growing population. Conventional agricultural practices are threatened by various setbacks such as harsh weather patterns, high operational costs, drought, insufficient land, etc. Hence, there should be a paradigm shift in the production of foods to ensure that there is an adequate supply of nutritious foods. Research is currently focused on the production of microbial-derived foods, in order to respond to these issues. Given its rich carbohydrate content, diverse applications, high accessibility, and ability to produce protein content that is comparable with meat- and plant-based protein, food waste has captured the interest of various stakeholders, including scientists, industries, and policymakers, in the quest for attaining sustainable protein technologies, as demonstrated in this review. Novel food waste biorefineries targeting SCPs will play a pivotal role in the advancement of alternative protein sources, particularly the development of 2G microbial protein. From a circular bio/economy standpoint, this strategy can unlock many economic opportunities, and lead to the concomitant production/recovery of diverse industrially relevant compounds (foods, biofuels, biomaterials, etc.), while contributing to environmental remediation. However, several bottlenecks must be addressed before the large-scale second-generation SCP processes can be realized, and these are discussed below.

**Figure 2:**
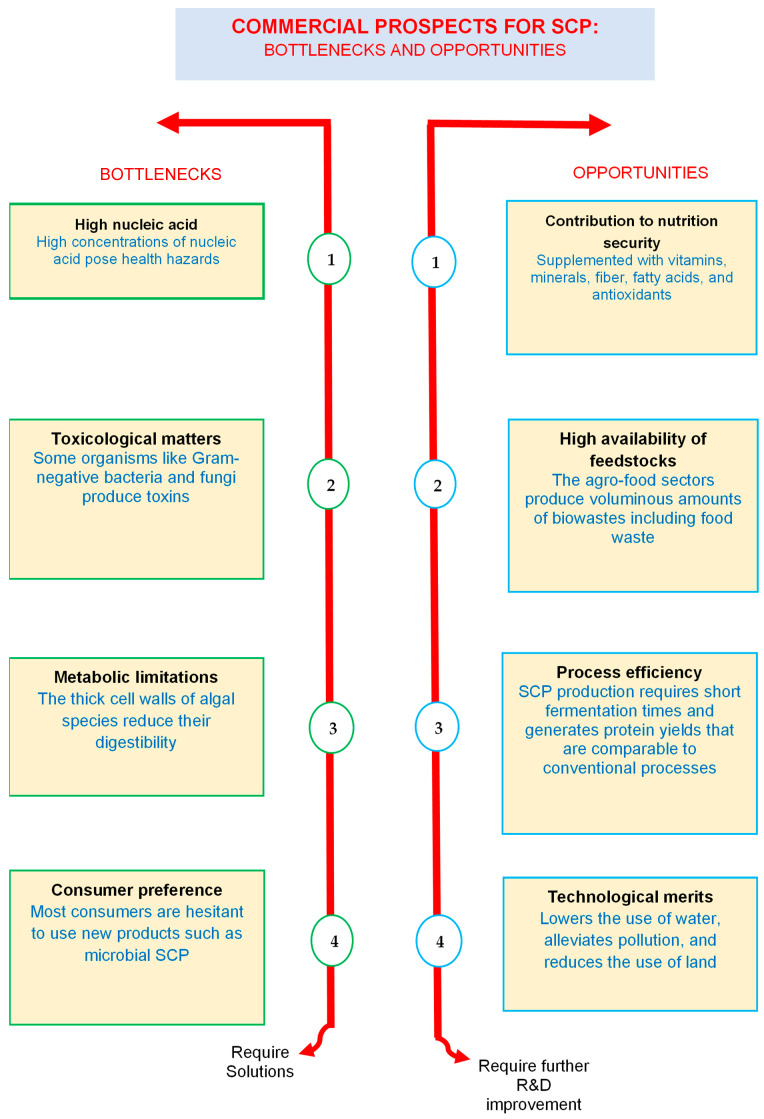
Bottlenecks and opportunities for commercial SCP [132].

- Most SCP studies are still conducted at bench-scale, implying that the actual process complexities are not fully understood in the literature. Therefore, scientific researchers must address this technological gap by conducting more pilot-scale demonstrations; this will lead to the large-scale implementation of SCP technologies, as evidenced by the formation of SCP-based companies that are exploring the use of different organic wastes as primary feedstocks for their upstream processes [17]. Innovations in “waste-to-protein” systems are also tailored toward the establishment of multi-product value chains, where SCPs are produced with other biobased products such as biopolymers, organic acids, biofertilizers, enzymes, etc., through a network of biorefinery pathways. Some of these studies have reached pilot-scale [130]. These biorefinery pathways could strengthen the economic potential of SCP processes, as well as reduce the downstream processing costs associated with SCPs. From an economic viewpoint, “waste-to-protein” innovations will spur a myriad of economic opportunities through the establishment of start-ups and large corporations specializing in second-generation SCP technologies, expand the protein production markets through sustainable approaches, create job opportunities, and strengthen the economic potential of food processing industries; such businesses can couple their sidestreams with SCP-producing biorefineries, leading to multifarious product chains and reduced costs associated with the disposal of effluents, as shown in Section 5.
- With the emergence of new scientific tools in bioprocessing, microbial ecology, and synthetic biology, scientists can use these advanced techniques to enhance the performance of biocatalysts by targeting the SCP-producing biochemical pathways. Ideally, these engineered strains should be able to withstand chemical inhibitors, reduce the fermentation periods, be applicable in biorefinery-based pathways, limit the proliferation of metabolites, and enhance the yields of SCPs. Although their individualistic effects are well known, is it also important to understand the synergistic interactions of the important operational setpoint parameters that are applicable during the upstream processing of SCPs, since this will help to obtain high yields. Such parameters include the pH, temperature, fermentation period, substrate concentration, etc.
- Although this is well documented in the literature, more toxicology and screening safety tests are still required to ensure that SCPs do not consist of contaminants, high RNA content, heavy metals, or pathogens, as these substances are unsafe for human and livestock consumption. This implies that SCPs must be subjected to vigorous downstream processes. Therein, several key steps are adopted to recover the SCP. These typically consist of biomass separation, decontamination, formulation, etc. The downstream process is also dependent on the inoculum source used during the fermentation process and its targeted use. For example, cell wall disruption is used in algal-based SCP production targeted for human consumption [155]. This is usually achieved through the use of mechanical, non-mechanical, or chemical routes. This involves mechanical mills, homogenizers, ultrasound equipment, microwaves, pulsed electric fields, enzymatic action, organic solvents, ionic liquids, or supercritical fluid extraction [156]. The method used for the disruption of algal cell walls needs to be evaluated, as it can affect the quality and quantity of protein [17].
- For second-generation protein-based technologies to reach industrialization, it is important for researchers from different scientific disciplines (e.g., molecular biology, microbial ecology, fermentation technology, chemical engineering, material sciences, etc.) to establish joint R&D collaborations to effectively address the technological barriers that prevent the scalability of these processes. Most importantly, it is necessary to include industries in these collaborations to conduct this technology at higher TRL levels (TRL ≥ 6). A more practical approach for food processing industries would be to create “cascade biorefinery” networks that will enable the use of their sidestreams for SCP production, thereby diversifying their product portfolio. This approach has been achieved by large biomass processing corporations [157].
- More studies involving the TEA assessment of SCP processes using food waste as a carbon source should be carried out in the literature, as this will provide deeper insights into the technical and economic feasibility of these processes. It is crucial to also conduct TEA studies when varying the inocula (bacteria, yeast, fungi, or algae), as this will enable scientists to have a broader perspective on the processes. TEA reports will help researchers and businesses make informed decisions regarding the most competitive SCP technologies that could be used as alternative protein sources.
- In addition to these suggestions, it is important to find markets that are willing to pay for the SCP products, and to establish regulations that promote biobased markets; this requires commitments from various stakeholders (scientific, industries, governments, etc.). Furthermore, SCP technologies can also leverage the phenomenon called “industrial symbiosis”, whereby the biowaste generated by agri-food industries can be supplied to SCP companies as a sustainable feedstock. European case studies showed that this is feasible, as it not only leads to a significant reduction in the amount of waste generated in the area, but also helps in terms of cost savings [158,159]. This concept has recently been adopted by some South African agro-food industries, whereby their waste streams are used by biowaste upcycling companies as a feedstock for their processes; this has resulted in a significant reduction in the costs associated with the treatment of these wastes, and the environmental burdens caused by their disposal [31]. However, to fast-track this concept, various barriers must be overcome, and these revolve around cooperation and trust amongst industries, the establishment of legislative frameworks to promote it, government incentives, etc.
- One of the main barriers facing second-generation SCPs is their full acceptance as a feed and food supplement, due to regulatory frameworks related to their safety and quality assurance. For SCP-derived products, the quality of the feedstock is crucial, as it can affect the nutritional composition and safety of the final SCP product [132]. Therefore, there are strict regulations regarding the manufacturing of alternative foods used for animal and human consumption in Western nations like the European Union, and this has led to the establishment of acceptable and unacceptable materials that can be used in SCP products [132]. Furthermore, the registration for novel foods in the EU requires a compilation of in-depth technical dossiers that demonstrate evidence for the safety of these products [160]. The EU’s Novel Food Regulation established a set of guidelines to be adhered to for the pre-market approval of novel foods [132]. Toxicological screening tests must be performed continuously by practitioners and industries to assess toxins and pathogens, as a means of conducting a safety assurance protocol before submitting their dossiers for consideration by food regulatory agencies. Developing countries such as South Africa could adopt similar frameworks, which will in turn promote the development of alternative foods while ensuring a high level of compliance. Despite this, the global market for SCP products is experiencing significant growth due to the increasing demand for healthier protein sources, resurging interest in microbial-driven technologies, and the adoption of technologies that align with the UN’s SDGs. In 2021, the SCP industry was estimated to be around USD 8 billion, and is anticipated to be around USD 18 billion by 2030, with an annual growth rate of 9–10% [161]. International trends show that SCP products will receive a tremendous boost in Asia, Europe, and North America; these trends are also supported by the agricultural sector, where there is increased pressure for the supply of alternative animal feeds [161]. Some of the bottlenecks that have been covered in this review, as well as the commercial opportunities for SCP technologies, are summarized in Figure 2.

## Data Availability

Not applicable.
